# Intestinal neuroendocrine tumor in a patient with pituitary adenoma. A case report and review of the current screening recommendations

**DOI:** 10.1186/1752-1947-1-140

**Published:** 2007-11-19

**Authors:** Cherif Boutros, Diana Cheng-Robles, Robert Goldenkranz

**Affiliations:** 1 Department of Surgery, Monmouth Medical Center 300 Second Avenue, Long Branch, NJ 07740, USA; 2 Department of Surgery, Newark Beth Israel Medical Center201 Lyons Avenue, Newark, NJ 07112, USA

## Abstract

**Introduction:**

Multiple endocrine neoplasia type 1 (MEN-1) patients are prone to develop carcinoid tumors. Few cases report the development of gastrointestinal carcinoid tumors in patients with MEN-1 syndrome related tumors. This is the first paper to report the occurrence of an intestinal carcinoid tumour in association with a pituitary adenoma.

**Case presentation:**

A **s**ixty eight year old female presented with intestinal obstruction four years after transphenoidal pituitary resection for pituitary adenoma. During surgical exploration and lysis of adhesions, we accidentally discovered an intestinal carcinoid tumour. Resection of the involved small bowel segment and the draining lymph nodes was undertaken. Postoperative follow up showed no biochemical or radiological evidence of residual tumor.

Neuroendocrine tumors (NETs) may occur as part of familial endocrine cancer syndromes including MEN-1. It is recommended that clinicians search thoroughly for MEN-1 in patients presented with NETs, however, there is no current consensus for screening patients suspected to have MEN-1 to rule out NET.

**Conclusion:**

We recommend screening patients suspected to have any familial type of endocrine tumors for the presence of NET.

## Introduction

Multiple endocrine neoplasia type 1 (MEN-1) is an autosomal dominant disease with a broad spectrum of clinical manifestations [1,2]. Though parathyroid hyperplasia, gastroenteropancreatic neoplasia, pituitary tumours and adrenal adenoma are the most frequent disease phenotypes [1,2], patients with MEN-1 are also liable to develop subcutaneous lipomas, gastrointestinal, bronchial, and thymic carcinoid tumours [3,4].

There are few case reports of the development of gastrointestinal carcinoid tumors in patients diagnosed with MEN-1 syndrome related tumors. Carcinoid tumor in these cases was located in the duodenum [5], and the stomach [6]. This is the first paper to report the occurrence of an intestinal carcinoid tumour in association of a pituitary adenoma.

## Case presentation

Our patient was a 68 year old African American female who developed diffuse abdominal pain, associated with nausea and bilious vomiting, 24 hours prior to her emergency room visit. The pain was localized to the epigastric region and had a progressive course. Her last bowel movement was reported as four days prior and she denied any recent weight loss, wheezing, flushing, palpitation or change in bowel habits.

The patient's past medical and surgical history included hypertension, hyperlipidemia, total abdominal hysterectomy and bilateral salpingoophorectomy secondary to fibroids.

The patient also reported a history of progressive loss of vision ten years earlier that was investigated by brain MRI after an extensive ophthalmological evaluation. The MRI showed a pituitary tumor and the patient benefited from a transphenoidal pituitary tumor resection. The pathological examination revealed a chromophobic pituitary adenoma.

The patient had a strong family history of cancer. Her father died from colon cancer, a brother died from esophageal cancer, an uncle died from brain tumor, one aunt had been diagnosed with breast cancer and one aunt with gastric cancer.

**Figure 1:**
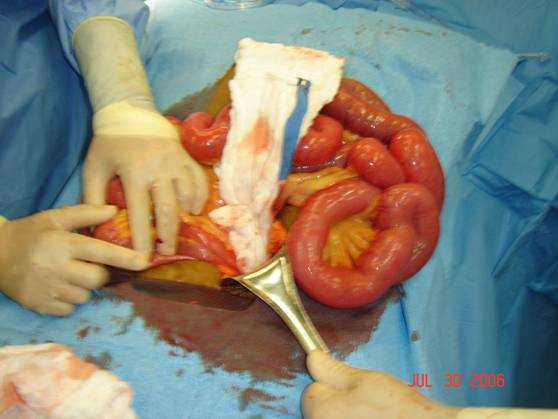
Small bowel serosal lesion.

**Figure 2:**
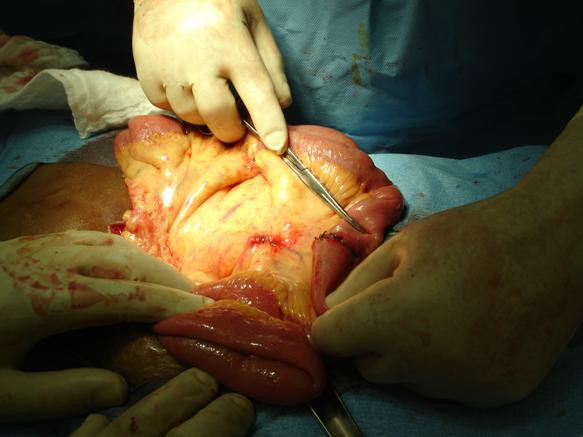
Resected serosal lesion and suspicious mesenteric lymph node.

Upon physical exam, the patient had a tense, distended abdomen, with a well healed paramedian incision and no bowel sounds. There was non localized diffuse tenderness with positive rebound and voluntary guarding. Rectal examination revealed no masses and an empty vault. Laboratory values revealed no leukocytosis, however the lactic acid level was elevated. A computed tomography scan of the abdomen revealed a small bowel obstruction. Subsequently, the patient was decompressed with a nasogastric tube and fluid resuscitation, and brought to the operating room for an exploratory laparotomy. Intraoperatively, there was significant small bowel congestion with no necrosis. One adhesive band was found and lysed at the mid jejunum where it was fixed to the pelvic wall. The bowel was thoroughly inspected to look for any other points of obstruction or abnormalities. A serosal lesion was found on the surface of the jejunum ten centimeters from the adhesion (Figure 1). Also a suspicious hard draining mesenteric lymph node was seen. The serosal lesion, and the suspicious lymph node were both resected (Figure 2), and sent for pathologic determination.

Postoperatively, the patient did well, however, the pathological evaluation of both the serosal lesion and the mesenteric lymph node revealed carcinoid tumor.

**Figure 3:**
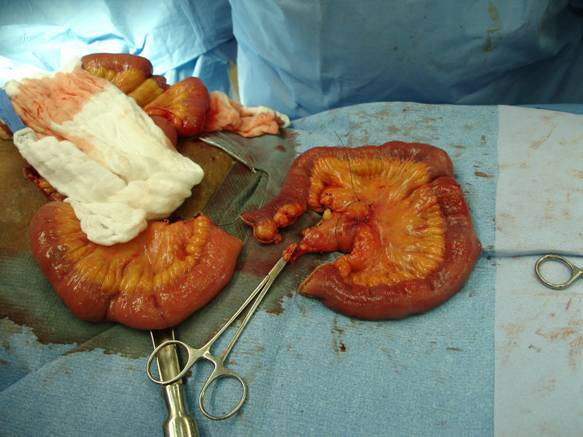
Small bowel resection segment.

Three days later, the patient was brought back to the operating room for exploratory laparotomy and small bowel resection. During the surgery, there was no intestinal lesion noted, and about 15 cm of small bowel on each side from the previous serosal lesion was resected with its corresponded mesentery (Figure 3). One enlarged and firm mesenteric lymph node, included in the specimen was marked with a stitch.

**Figure 4:**
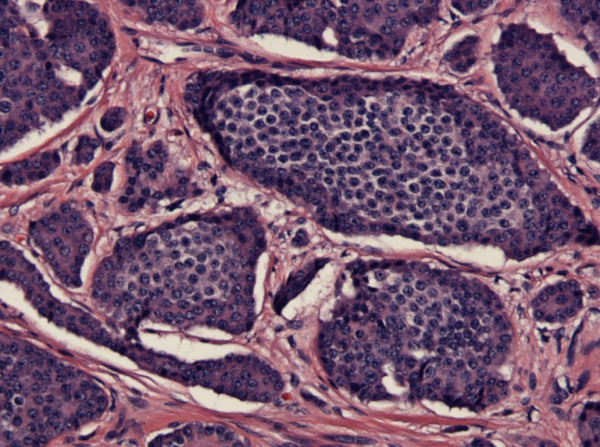
Small Bowel with H&E staining × 20 magnification: Carcinoid tumor penetrating the subserosa.

**Figure 5:**
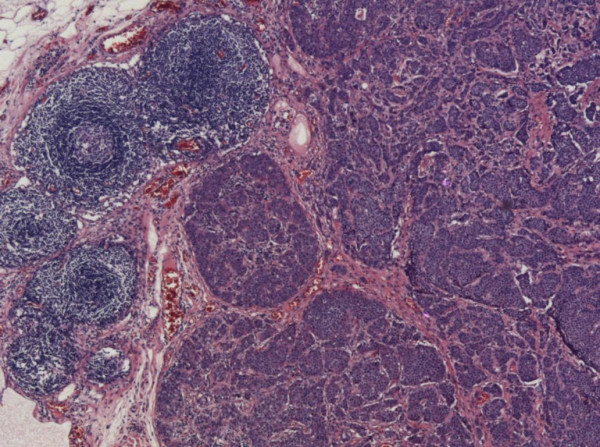
Lymph node with H&E staining ×4 magnification: Carcinoid metastases.

The pathological examination of the specimen revealed a carcinoid tumor approximately 0.4 cm in greatest dimension, penetrating subserosa five centimeters from the previously resected serosal lesion (Figure 4). A metastatic carcinoid tumor was seen in three out of 17 lymph nodes (Figure 5) including the one marked with the stitch. The surgical resection margins were negative.

During the postoperative inpatient period, a 24 hour urine 5-Hydroxyindole Acetic Acid (5-HIAA) was within normal value and no focal area of increase uptake was noted on an octreotide scan.

There was no postoperative morbidity, and the patient was followed as an outpatient at two and six month interval. During these follow up visits, the patient reported feeling better and denied any weight loss, wheezing, flushing, palpitations or diarrhea.

A postoperative CT scan of the chest abdomen and pelvis six months after the surgery, revealed no evidence of recurrent disease, and no intra abdominal masses. A repeat octreotide scan at six months after the surgery did not show any area of increase uptake. Chromogranin A level was followed, and was decreasing from 142 ng/ml at two months post resection, to 64 ng/ml at six months post resection.

## Discussion

Neuroendocrine tumors (NETs) constitute a heterogeneous group of neoplasms with common characteristics and biological features. NETs were previously referred as apudomas, by Pearse in 1969 [7], when he described peptide-secreting endocrine cells that share an ability to take up precursors of biologically active amines, to produce active amine through subsequent intracellular decarboxylation, hence APUD (amine precursor uptake and decarboxylation).

This widespread phenomenon initially reported by Pearse himself in 1964, when describing the calcitonin-secreting parafollicular C cells of dog thyroid, was also found in other peptide hormone-secreting cells, for example in the corticotrophs of the anterior pituitary gland and in the b-cells of the endocrine pancreas. Currently, APUD cells including more than 40 cell types are best referred as a diffuse neuroendocrine system.

Primary gastroenteropancreatic NETs can be asymptomatic but may present with obstructive symptoms, and carcinoid syndrome when tumors metastases to the liver with the subsequent release of vasoactive substances responsible of the classical picture of flushing, diarrhea, lacrimation, palpitations, and wheezing.

Midgut carcinoid tumours have a wide range of presenting signs including abdominal pain, nausea and vomiting, weight loss, diarrhea, gastrointestinal bleeding and carcinoid syndrome. However, significant number are incidentally discovered in resected surgical specimens when surgery had been undertaken for other intra abdominal disease [8].

Though the majority of NETs occur spontaneously, NETs may occur also as part of complex familial endocrine cancer syndromes such as MEN-1, MEN-2, Neurofibromatosis type 1(NFT type 1), Von Hippel Lindau disease, and Carney's Complex [9-11].

The incidence of MEN-1 in gastroenteropancreatic NETs was reported to range from 30% in gastrinoma to virtually none in gut carcinoid [12]. However, it is recommended to search thoroughly for MEN-1, MEN-2 and NF1 in all patients presented with NETs by obtaining family history, clinical examination, appropriate biochemical and radiological investigations and genetic testing [13].

At the same time, surveillance for persons known to have MEN-1 to rule out NET was previously suggested [14]. The current follow up recommendations for patients known to have MEN-1 syndrome start at age of ten with yearly physical examination, laboratory tests including fasting serum gastrin, calcium, albumin, pancreatic polypeptide levels, and abdominal CT or MRI. Every other year these patients should also have a head MRI and prolactin level [14,15]. Currently, screening for NETs is not mandatory in these patients.

For persons suspected to have MEN-1, including those with a first degree affected relative but without gene testing, and members of a clinical group at risk, the current recommendations do not include screening for NETs [14].

This case report is the first to report the occurrence of an intestinal NET in a patient previously documented to have a pituitary adenoma. In our patient, intestinal NET was found in a specimen taken during abdominal surgery for other reason. Surgical resection of the tumor with postoperative negative octreotide scan, decreasing chromogranin A level, and 5-HIAA within normal values reflects a favorable prognosis.

We believe that screening patients suspected to have any familial type of endocrine tumors for NETs, could help early diagnosis in asymptomatic patients, and offer them the best chance for curative resection. The screening protocol can possibly include biochemical tests including 5-HIAA, chromogranin A, radiological evaluation including an octreotide scan, and endoscopic examination including endoscopic ultrasound. Screening large numbers of patients with either documented or suspected MEN-1 syndrome using biochemical, radiological and endoscopic tests will help to select the best, and the most reliable screening modality. A follow up of these patients could show a potential beneficial effect related to the early detection of NETs diagnosed by this screening protocol.

## Conclusion

A critical component in the diagnosis of a neuroendocrine tumor is suspicion of its presence. We believe that screening patients suspected of having any familial type of endocrine tumors for NETs could help early diagnosis in asymptomatic patients and offers these patients the best chance to have a curative resection. Further studies are required to investigate the best screening modality.

## Competing interests

The author(s) declare that they have no competing interests.

## Authors' contributions

Study conception and design: CB and RG were responsible for the study conception and design. DCR and CB acquired the data and drafted the manuscript. CB RG and DCR critically revised the manuscript. All authors read and approved the final manuscript.

## Consent

Written informed patient consent was obtained for publication
